# Effect of Iron Status in Rats on the Absorption of Metal Ions from Plant Ferritin

**DOI:** 10.1007/s11130-014-0413-1

**Published:** 2014-04-12

**Authors:** Magdalena Zielińska-Dawidziak, Iwona Hertig, Halina Staniek, Dorota Piasecka-Kwiatkowska, Krzysztof W. Nowak

**Affiliations:** 1 Department of Food Biochemistry and Analysis, Poznań University of Life Sciences, Mazowiecka 48, 60-623 Poznań, Poland; 2 Department of Animal Physiology and Biochemistry, Poznań University of Life Sciences, Wołyńska 35, 60-637 Poznań, Poland; 3 Department of Human Nutrition and Hygiene, Poznan University of Life Sciences, Wojska Polskiego 31, 60-624 Poznań, Poland

**Keywords:** Sprouted soybean seeds, Lead-ferritin, Iron status, Iron-deficient rats

## Abstract

An isolate of lead-ferritin obtained from soybean seeds sprouted in 25 mM of PbNO_3_ was introduced into the diet of both iron-deficient and iron non-deficient male rats. After a 21-day administration period, statistical differences in the lead accumulation in the femurs of the rats were noted. Iron-deficient rats accumulated more than four times the amount of lead in their bones than rats without iron-deficiency. No further decrease was observed in haemoglobin concentrations in the groups of animals fed with lead isolates, either iron-deficient or iron non-deficient. Also, no differences in the mean corpuscular haemoglobin (MCH) and mean corpuscular volume (MCV) were observed at the end of the experiment in the group of iron non-deficient rats fed with lead-ferritin isolate compared to the control group of iron non-deficient rats. In the iron-deficient group fed with lead-ferritin isolate, a small increase in haemoglobin concentrations, MCH, MCV and mean corpuscular haemoglobin concentrations (MCHC) was recorded. The results presented in this paper confirm that lead from the tested preparation—lead ferritin isolate—was better absorbed by those rats with induced iron deficiency anaemia. Additionally, we may also suspect based on the obtained results that absorption of ferritin-iron depends on iron status in the body.

**Electronic supplementary material:**

The online version of this article (doi:10.1007/s11130-014-0413-1) contains supplementary material, which is available to authorized users.

## Introduction

Iron deficiency anaemia is a very common nutritional disorder in humans. Currently, a good source of this microelement with a better bioavailability profile is required. Experiments are focused especially on non-hem iron, improvement of its bioavailability, reducing restrictive factors, recognizing the mechanisms of its absorption and the side-effects of new iron sources introduced into the diet or supplements [1–6].

One of the method being considered for fortification of the human diet with iron is food enrichment in plant ferritin. Ferritin delivers iron to the body in an oxidized Fe^3+^ form, enclosed in a peptide shell. Therefore, it is a safe source of iron. A new interesting source of plant ferritin are sprouted soybean seeds, which are biofortified during their growth [6]. Experiments on ferritin iron bioavailability are at this moment inconsistent. Some previous reports suggested its limited absorption by the human body [2], while present studies indicate on high bioavailability of ferritin iron [3, 5]. Moreover, the resistance of ferritin to digestion is still being discussed [5, 7, 8].

Iron absorption usually increases 10-fold in the case of its deficiency and this rule applies both to heme and non-heme iron. The mechanism of soybean ferritin absorption is different than for Fe(II) ions and is closer to heme absorption. Ferritin is also uptaken via endocytosis where another receptor (assembly protein 2 complex subunit mu) is involved in this process [9]. The high concentration of iron in the shell of ferritin (up to 4,500 of iron atoms per molecule) and the transport system suggest, that it may be easy to exceed the supply of iron in an organism during the supplementation of food with extremely large doses of ferritin. Thus, the purpose of the present research was to verify the hypothesis that the absorption of iron accumulated in the apoferritin depends on the iron status in the body, if it is strongly regulated and inhibited in organisms which do not suffer from iron deficiency anaemia. The experimental model in the paper was based on information that iron competes with lead in the gut and that there is strong correlation between lead and iron absorption [10]. Our model assumed the introduction of lead ions into the ferritin shell, and then, after the period of feeding the rats, comparing the lead concentrations in the tissues of animals with iron deficiency anaemia and non-iron deficient.

A protein isolate derived from a plant, which has grown in the presence of high lead concentrations, can be a good source of ferritin filled with lead instead of iron. The substances involved in lead neutralization in plants are well characterized. They are bound in a variety of molecules that are intended to neutralize their harmful effects on plant physiology. The first group of these molecules contains low molecular weight compounds, such as citrate, oxalic acid, malic acid, phosphorus compounds, and especially phytin and some amino acids and their derivatives [11].

The next group includes peptides, which are characterized by a low molecular weight (600–4,000 kDa), thermal stability and a high content of glutamic acid and cysteine. This group comprises:

- Glycine phytochelatins (γ-Glu-Cys)*n*-Gly, (where ‘*n*’ has a value from 2–11) [12]
- Alanine homo-phytochelatins—peptides commonly occurring in legumes with the formula (γ-Glu-Cys)*n*β-Ala (where ‘*n*’ has a value from 2–7) [13]
- Other phytochelatin homologs—peptide types (γ-Glu-Cys)_2_Glu, (γ-Glu-Cys)*n* and (γ-Glu-Cys)*n*Ser [14, 15]
- Glutathione [13].

Another group can be represented by metalothionein, low molecular weight (6–7 kDa) and heat stable proteins with repeated sequences Cys-XX-Cys or Cys-Cys-X (where X is usually Lys, Ser or Arg) [14]. A further group contains low molecular weight heat shock proteins [15, 16]. Finally, there is phytoferritin, which is a large protein with a molecular weight of 480–600 kDa [17], and the other vegetable proteins induced by heavy metals (principally cadmium) with unrecognized functions, such as a protein complex (18 kDa) induced in rice seedlings [18] or proteins from cultures of carrot root hair (30 kDa and 35 kDa) [19].

## Materials and Methods

### Soybean Lead-Ferritin Isolate Preparation

Soybean (*Glycine max*, Naviko var.) from the Department of Genetics and Plant Breeding, Poznan University of Life Sciences was used to achieve the planned research tasks. The seeds were immersed every day over 7 days for a period of two hours in solutions of 0–25 mM PbNO_3_, under conditions of daylight illumination.

Seeds germinated in 25 mM PbNO_3_ (both radicle and cotyledons) were subjected to a homogenization process in 20 mM Tris–HCl with 12 mM NaCl buffer (pH = 8.0). Then, ferritin isolation via the salting out method was performed according to Smól [20]. In addition, the resultant isolates were twice filtered on a ceramic filter (cut off: 100 kDa, Helicon S10), to separate off both proteins other than ferritin capable of lead binding as well as low molecular lead compounds. Both protein concentration (with Bradford assay, [21]) and lead content in the filtered isolates were determined.

### Lead Determination

Weighted amounts of samples (~1 g for kidneys and diet, 1–2 g of bones) were mineralized in spectrally pure 65 % HNO_3_ (Merck) using a MARS-5 microwave (CEM). Lead content was determined using flame atomic absorption spectrometry, at a wavelength of 217.0 nm and a gap width of 0.3 nm (AAS Carl-Zeiss-3).

### Determination of Iron in the Diet

Approximately 1-g of the diet sample was wet mineralized as described above for lead. The iron content in these mineralizates was determined using the same spectrometer at a wavelength of 248.3 nm and a gap width of 0.15 nm.

### The Study of Lead-Ferritin Absorption

The experiment performed for analysis of the availability of lead from ferritin was conducted on groups of rats either with induced iron-deficiency anemia or non iron-deficient. Male Wistar rats from the breeding centre in Brwinów, with a balanced initial body weight of 180 g ± 10 g, were kept in the vivarium of the Department of Animal Physiology and Biochemistry at Poznan University of Life Sciences. The animals were assured appropriate conditions (a temperature of 20–22 °C, relative air humidity at the level of 55–60 %, and optimum daily lighting cycle, i.e. 12 h of light/12 h of darkness). They were placed in collective cages (2–3 individuals in a cage), fed *ad libitum* with suitable diets (depending on the experimental stage and the group the particular individuals were assigned to) and supplied with deionized water in order to eliminate additional iron sources.

At the first stage of the experiment, the animals were divided into two groups. The first group (the iron-deficient) was fed with a diet low in iron, prepared according to AIN standards [22], eliminating iron citrate (III) from it. The iron content in the diet was decreased to 6.99 ± 1.14 mg Fe/kg. The other group (the non-deficient) was fed with a diet containing optimum iron content for feeding rats, *i.e.*, 53 ± 5.2 mg Fe/kg [22].

This stage of the experiment was continued up to the moment, when the initial hemoglobin level determined in the blood from the tail in the iron-deficient group decreased to the value of ~9.47 g/dl. Then, the second stage of the experiment started and the animals were divided into the final experimental groups:

D: Iron-deficient control group, fed with a diet low in iron for the whole experimental period (first and second stage),
DPb: Iron-deficient group administered with lead-ferritin during the second stage,
NPb: Non-deficient group fed with lead-ferritin during the second stage,
N: Non-deficient control group, where animals received a diet ensuring an optimum iron supply for the whole experimental period (first and second stage).

The groups were composed of 8 individuals. The lead-ferritin isolates were introduced to the diet of the animals from the DPb and NPb groups. The diets containing lead isolates were prepared by mixing 11 kg of a proper diet, i.e. 11 kg diet low in iron with 12.5 l lead isolates or 11 kg diet with an optimal content of iron with the same amount of isolates. The resulting blend was dried at 40 °C, stirring frequently. The amount of 13.4 kg of the diet with a lead content of 33.4 ± 3.1 mg/kg, and an iron content of 7.8 ± 0.2 mg/kg was obtained.

This second stage of the experiment lasted 21 days. At the end, the animals were sacrificed and their organs (liver, spleen, heart and kidneys), blood and femurs were separated. Downloaded organs were weighed and prepared for further analysis.

## Results and Discussion

From the division given in the introduction it may be concluded that only one extremely high molecular weight substance is present in the group (i.e. plant ferritin). In the presented experiment, it was exploited the capacity of plant ferritin to bind lead from the experimental medium. Ferritin isolation and subsequent filtration guaranteed to have depleted all other substances which could contain lead in their structure.

**Fig. 1:**
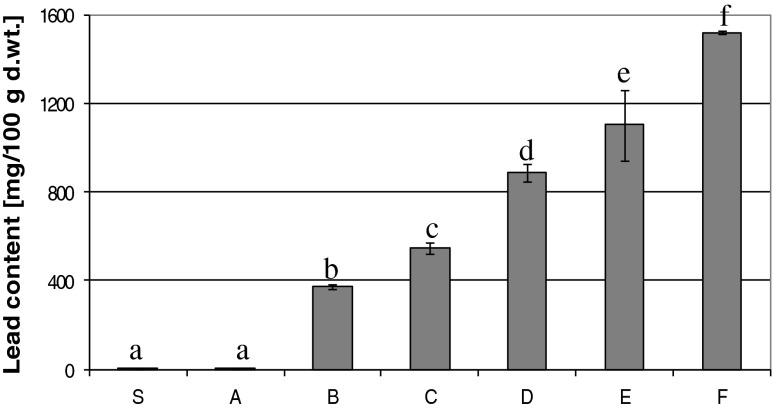
The content of lead in soybean seeds (S) and sprouts obtained after seven days of cultivation, respectively in solutions of PbNO_3_ with concentrations: A - 0, B - 5, C - 10, D - 15, E - 20 or F - 25 mM. Different letters show statistically significant differences at *P* < 0.05

**Fig. 2:**
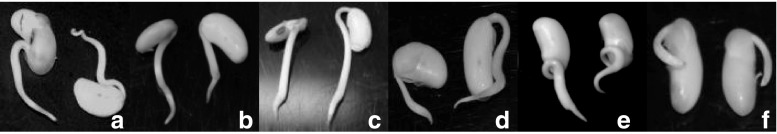
Photographs of sprouted soybean seeds obtained in the different concentrations of PbNO_3_: **a** - 0 mM (water), **b** - 5 mM, **c** - 10 mM, **d** - 15 mM, **e** - 20 mM, **f** - 25 mM

The material chosen for the preparation of lead-ferritin were germinated soybean seeds. Soybean seemed to be the most resistant among the examined plants to the presence of lead in the culture medium (i.e. soybean, lentil, lupin and wheat) (data not presented). Soybean seeds germinated at a concentration of 25 mM PbNO_3_, accumulating lead in the amount of 1.52 ± 0.008 g/100 g d.m. (dry matter). Figures 1 presents the lead content in the sprouted soybean seeds depending on lead concentration in the growth medium. It must be noted here that hardly any changes in the appearance of sprouts were observed (Fig. 2), in contrast to the sprouts cultured with FeSO_4_ [23]. The thickening of sprouts, their twisting and shortening was observed only in highly concentrated solutions of PbNO_3_, compared to seedlings grown in ordinary conditions (*i.e.*, in water). This is a phenomenon very dangerous to human health, because lead-rich sprouted seeds are indistinguishable from those normally obtained. This indicates a clear need to control the chemical purity of the germination media during cultivation of seeds. The accumulation of beryllium, aluminum, zinc, cadmium and lead in ferritin was confirmed [17, 20, 24, 25]. Concentration of lead in germinated soybean seeds was very high. It was observed that in experimental conditions seeds sprouted with the highest concentration of PbNO_3_ accumulated ~1,800 times more lead than was contained in the dry seeds.

Ferritin isolates were prepared according to the methodology developed for the isolation of lupin ferritin [20], which was based on the selective salting out of globulins resistant to temperatures up to 70 °C and pH > 4. Additionally, in order to remove small molecules, which may contain lead in their composition, filtration was performed with a ceramic filter (with a cut off 100 kDa). Ferritin is the only lead-chelating compound with MW significantly above 100 kDa.

The lead concentration determined in the permeate was about 11 % of that remaining in the retentate. Some further losses were also related to the residue of lead compounds in the filter pores. These losses differed depending on the volume of filtered isolate and amounted to as much as 50 %. Additionally, the protein level in the permeate, as determined with the Bradford method [21], was approximately 20-fold lower than in the retentate. The obtained isolates were used for the preparation of rat diets.

Discussion of ferritin bioavailability is extremely complex, and includes both the problem of ferritin stability in the gastrointestinal tract, as well as the analysis of the mechanisms and control of its absorption [5, 9, 26]. The plant ferritin absorption profile for the ferritin isolates and powdered, dried sprouts has been presented previously in comparison to FeSO_4_, standard pharmaceutical dosage [6]. The absorption of these three iron forms (ferritin isolate, powdered sprouts and FeSO_4_) during this parallel experiment was comparable (results are attached in the supplementary materials). However, that experiment [6] did not provide any information about the ferritin iron absorption by those animals without iron deficiency. Because of the high concentration of iron in the powdered soybean sprouts (up to as much as 750 mg/100 g d.wt.), the problem of ferritin iron absorption regulatory mechanisms by the non iron-deficient organism seems to be more noteworthy. One absorbed molecule of the protein transported through intestine enterocytes introduces as many as 4,500 atoms of iron into the blood. If the regulation of ferritin iron absorption is not properly efficient, the increased supply of this form of iron in food or in dietary supplements may pose a serious threat to the health of the consumer. The main danger is over-accumulation of iron in the body.

Therefore, the following question arises as to whether the absorption of a high dosage of phytoferritin from the diet is dependent on the iron status in the rat bodies. A properly balanced rat diet includes different sources of iron, but it is very difficult to exclude any other source of iron. Due to the relationship of iron and lead metabolism [10], the decision was made to control the supply of ferritin filled with lead and analyze its accumulation in rat tissues. The lead ferritin filtered isolate was administered to both the rats deficient in iron (DPb group) and also those with a proper iron status (NPb group). Two control groups consisting of groups N and D were additionally tested during the whole experiment. Group N included animals non-deficient in iron that were fed with a standard diet ensuring optimum iron supply. Group D included rats which received a diet low in iron (6.99 ± 1.14 mg Fe/kg of diet).

It was assumed that, if iron deficient animals accumulated more lead than healthy animals in their tissues (bone and kidney), this would confirm the thesis by Murray-Kolb et al. [3, 26] that the absorption of iron from ferritin is dependent on the degree of iron saturation in the body.

After the first stage of the experiment, the difference in the level of hemoglobin in the animals’ blood (HGB1) differed statistically significantly between the iron-deficient and non deficient subjects (Table 1). For the further stages of the experiment, animals were divided into four groups based on HGB1 concentration.

**Table 1:** Some of the morphological parameters of the experimental animals blood. Group of animals: iron deficient (D) iron deficient fed with leadferritin isolates (DPb) iron non-deficient fed with lead-ferritin isolates (NPb), iron non-deficient (N)

Group of animals	HGB (1)* [g/dl]	HGB (2)** [g/dl]	HCT [%]	MCV [fl]	MCH [pg]	MCHC [g/dl]
D	9.47 ± 0.83^a^	9.09 ± 0.77^a^	38.32 ± 3.11^b^	28.97 ± 2.34^a^	9.75 ± 0.84^a^	20.17 ± 0.26^a^
DPb	9.47 ± 0.42^a^	10.02 ± 0.52^b^	30.12 ± 1.95^a^	34.19 ± 2.25^b^	11.38 ± 0.75^b^	22.32 ± 0.47^b^
NPb	13.76 ± 1.01^b^	13.11 ± 0.29^c^	39.32 ± 1.71^b^	45.73 ± 1.83^c^	15.28 ± 0.74^c^	28.39 ± 0.79^c^
N	13.87 ± 0.27^b^	13.75 ± 0.67^c^	39.61 ± 1.90^b^	46.78 ± 1.09^c^	15.52 ± 0.34^c^	29.52 ± 0.21^d^

* *HGB* after the first stage of the experiment, ** *HGB* at the end of the experiment

Different letters in a column show statistically significant differences at *P* < 0.05

**Fig. 3:**
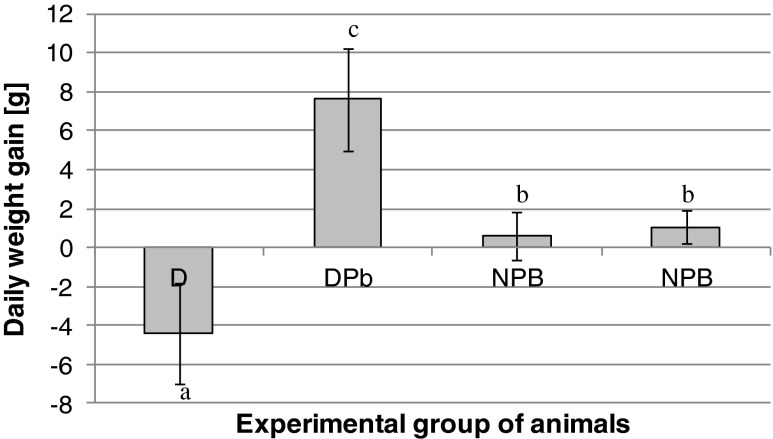
Daily weight gain of experimental animals. Group of animals: D - iron deficient, DPb - iron-deficient fed with lead-ferritin isolates, NPb - iron non-deficient fed with lead-ferritin isolates, N - iron non-deficient. Different letters show statistically significant differences at *P* < 0.05

The second stage of the experiment (feeding the animals with lead-ferritin) lasted for 21 days. The short duration of the experiment was dictated by the poor condition of the rats, due to the long period of being fed with a diet low in iron. The problem has been discussed previously [6] and it must be emphasized that the worst health status was observed in D group, while the condition of the other animals was satisfactory. The problem encountered by animals from D group resulted from a progressive decline in HGB concentrations (Table 1). During the second stage of the experiment, a continuous decrease in the body weight of these animals occurred, while the largest daily weight gain was observed for iron deficient animals treated with lead-ferritin isolate (Fig. 3). This finding was probably associated with an increased in dietary intake by the test specimens.

At the end of the experiment, the haemoglobin concentration (HGB2) in iron-deficient animals (D group) was the lowest and differed statistically from the remaining groups of animals. The increased concentration of haemoglobin in the group of iron-deficient animals, which received lead-ferritin isolate (DPb group), is due to a slight increase in the supply of iron in the diet (~0.81 mg/kg of the diet, *i.e.*, ~11.5 %) as well as increased consumption. However, no significant changes between HGB1 and HGB2 were observed within each experimental group. It may be also concluded that the absorption of lead did not affect the degradation of haemoglobin in the blood of rats, probably due to the short duration of the experiment.

The other studied parameters concerning the condition of studied animals are in accordance with expectations. This applies both to controlled morphological parameters (Table 1) and organ weights (Table 2).

**Table 2:** Organs size of experimental animals. Group of animals: iron deficient (D), iron deficient fed with lead-ferritin isolates (DPb), iron non-deficient fed with lead-ferritin isolates (NPb), iron non-deficient (N)

Experimental group of animals	Liver weight (% body mass)	Kidneys weight (% body mass)	Spleen weight (% body mass)	Heart weight (% body mass)
D	2.39 ± 0.17^a^	0.62 ± 0.07^c^	0.14 ± 0.02^a^	0.30 ± 0.04^b^
DPb	2.39 ± 0.27^a^	0.56 ± 0.05^b,c^	0.12 ± 0.02^a^	0.29 ± 0.03^b^
NPb	2.80 ± 0.43^a^	0.51 ± 0.04^a,b^	0.13 ± 0.02^a^	0.28 ± 0.02^a,b^
N	2.65 ± 0.22^a^	0.45 ± 0.03^a^	0.13 ± 0.03^a^	0.24 ± 0.02^a^

Different letters in a column show statistically significant differences at *P* < 0.05

Among the parameters associated with blood counts, i.e. MCV, MCH and MCHC, the lowest average values are observed for animals fed with a low iron diet throughout the duration of the whole experiment (D group), and the highest for animals fed with AIN diet (N group). A small increase in the MCV, MCH and MCHC levels in the DPb group might also be explained by the increase in the content of iron in the diet with lead isolate. The lowest hematocrit level is observed in the DPb group (iron-deficient, treated with Pb-isolate). Since this group showed an increase in the value of MCV, it may indicate an increase in the plasma volume. A similar result was observed in animals previously treated with iron-ferritin isolate [6, 20].

Extremely important is the fact that almost all the analysed blood parameters (HGB2, HCT, MCV and MCH) of animals without iron deficiency are at the same level; no differences were observed in their levels for those animals fed with a diet either containing or not containing lead. Considering the fact that 99 % of lead after absorption is bound to the erythrocytes and that serum lead half-life is longer than the duration of experiment [27, 28], this is the first confirmation that the absorption of metal ions from ferritin by organisms with proper iron status is inhibited. Usually, lead toxicity is connected with the development of anaemia [28]; however, this was not seen in the DPb group.

No statistical differences were observed in the ratio of liver weight to body weight (%LW) of the experimental rats (Table 2). This effect had been previously noted by Zielińska-Dawidziak et al. [6]. In iron deficient organisms both a significant reduction in the liver mass and volume, which is typical for iron deficiency [6, 29], as well as in the total body weight took place. Therefore, the ratio of the organ weight to the body weight remained the same. There were no differences in the %LW among all the tested animals. Even though there were no changes in the fatness of the studied livers, the pale colour of the livers from rats belonging to D and DPb groups was visible.

In contrast to the liver, the percentage of kidney weight (%KM) of rats from D and N groups significantly differed. This effect, also noted by Rothenbacher and Sherman [30], resulted from shrunken renal glomeruli and/or changes in its hypercellular structure. Homogenous groups, in terms of %KW, are formed from iron-deficient rats (both D and DPb), non iron-deficient rats (N and NPb) and also rats fed with a lead-rich diet (DPb and NPb). Forming the last homogenous group is obvious, if you consider the role of the kidney in the metabolism of lead [31].

Iron deficiency has no effect on spleen weight as a percentage of body weight (%SW) [32]. The short time of the experiment probably influenced the fact that there was no significant change in the weight of these organs obtained from animals fed with the diets containing lead.

The increase in the heart weight of iron deficient animals (from D and DPb group) is a common result occurring cardiomegaly [30]. Additionally, a homogenous group is formed by rats from D group, as well as rats from DPb and NPb groups. Heart hypertrophy in response to the toxic effects of lead is usually observed in animal experiments, even after application with low doses of the metal during feeding [32].

The concentration of lead in the bones and kidneys is evidence of the bioavailability of lead [31]. Despite the fact that the initial accumulation of lead occurs in the kidneys, the concentration of Pb in these organs was below the level of quantification. However, the presence of lead was detected in the bones of the animals.

**Fig. 4:**
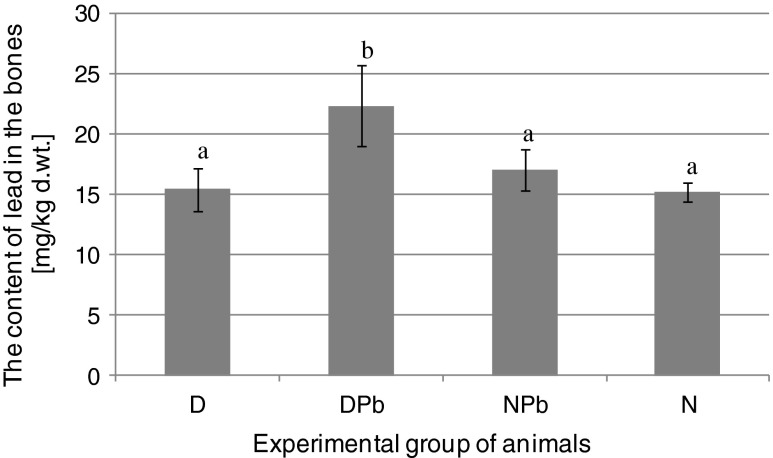
The content of lead in the bones of rats after 21 days of experiment. Group of animals: iron deficient (D), iron deficient fed with lead-ferritin isolates (DPb), iron non-deficient fed with lead-ferritin isolates(NPb), iron non-deficient (N). Different letters show statistically significant differences at *P* < 0.05

At the end of the experiment the lead level in the femurs of animals with iron deficiency treated with lead-ferritin isolate differed significantly not only from the control groups (N and D), but also from the group of animals with the proper iron status, which were administered with lead-ferritin (Fig. 4). The lead level in the bones of the animals from the DPb group increased by ~ 45 % compared to the level of lead in the control groups with induced anaemia (D) and without iron deficiency (N). The results confirm that the absorption of lead from ferritin is dependent on the state of the body’s iron saturation. In a group of non iron-deficient animals, treated with a lead preparation of ferritin (NPb), the increase in lead content in the bones was about 11 %. It was more than four times less than in the case of the DPb group. The differences between the concentrations of lead in the bones of D, N and NPb were statistically insignificant; although, we may assume that is a result of the short duration of the experiment. The observed trend should suggest that metal ions enclosed in the shell of ferritin were absorbed less efficiently, when the iron status of the test animals is proper.

The analysis of lead accumulation in femurs of iron-deficient and iron non-deficient rats after their feeding with lead-ferritin isolate confirmed that decreased iron status increases the absorption of the metal ions from lead-ferritin isolates.

On the basis of the presented results, it should be expected that absorption of iron ions enclosed in the ferritin strongly depends on the iron status of the organism being fed. Thus, excessive metal accumulation from plant ferritin is subjected to effective regulation in the digestive tract. The inhibition of ferritin absorption by the organism with proper iron status needs further explanation because it is difficult to suggest whether iron absorption by the enterocytes or the release from lysosomes is limited at this stage. Moreover, even if the stability of ferritin in the digestive process is still under discussion, the present results are additional confirmation for the controlled absorption of the metal ions from plant ferritin.

## Electronic supplementary material

Below is the link to the electronic supplementary material.

Electronic supplementary material Fig. 1 The changes in hemoglobin (HGB) concentration after supplementation of iron-deficient rat with ferritin-iron. The chart presents the hemoglobin concentration before (HGB1) and at the end (HGB2) of the experiment. Experimental group: ‘sprouts’—iron deficient rats supplemented with soybean sprouts enriched in ferritin iron; ‘isolate’—iron deficient rats supplemented with isolate of plant ferritin, ‘FeSO_4_’—iron-deficient rats supplemented with pharmaceutical preparation—FeSO_4_, ‘anemic’—control group of iron deficient rats during the whole experiment, ‘non-anemic’—control group of healthy animals. *Published in: Food Chemistry, 2012, 135:2622–2627; M. Zielińska-Dawidziak, I. Hertig, D. Piasecka-Kwiatkowska, H. Staniek, K.W. Nowak, T. Twardowski: Study on iron availability from prepared soybean sprouts using iron-deficient rat model* (DOC 57.0 kb)

Electronic supplementary material Table 1 Comparison of iron reserves in the liver and blood of experimental animals after supplementation with plant ferritin. Group of animals: iron deficient rats supplemented with soybean sprouts enriched in ferritin iron (1), iron deficient rats supplemented with isolate of plant ferritin (2), iron-deficient rats supplemented with pharmaceutical preparation (3), control group of iron deficient rats during the whole experiment (4), control group of healthy animals (5). *Published in: Food Chemistry, 2012, 135:2622–2627; M. Zielińska-Dawidziak, I. Hertig, D. Piasecka-Kwiatkowska, H. Staniek, K.W. Nowak, T. Twardowski: Study on iron availability from prepared soybean sprouts using iron-deficient rat model* (DOC 36.5 kb)

## Abbreviations

HGB: Haemoglobin concentration
MCH: Mean corpuscular haemoglobin
MCHC: Mean corpuscular haemoglobin concentrations
MCV: Mean corpuscular volume
