# Supplementary material for: Effect of Iron Status in Rats on the Absorption of Metal Ions from Plant Ferritin
Source: Plant Foods Hum Nutr. 2014 Apr 12;69(2):101–7. doi: 10.1007/s11130-014-0413-1 (PMC4032463; doi:10.1007/s11130-014-0413-1)
Supplement: Supplementary file 1 — The changes in hemoglobin (HGB) concentration after supplementation of iron-deficient rat with ferritin-iron. The chart presents the hemoglobin concentration before (HGB1) and at the end (HGB2) of the experiment. Experimental group: ‘sprouts’—iron deficient rats supplemented with soybean sprouts enriched in ferritin iron; ‘isolate’—iron deficient rats supplemented with isolate of plant ferritin, ‘FeSO4’—iron-deficient rats supplemented with pharmaceutical preparation—FeSO4, ‘anemic’—control group of iron deficient rats during the whole experiment, ‘non-anemic’—control group of healthy animals. Published in: Food Chemistry, 2012, 135:2622–2627; M. Zielińska-Dawidziak, I. Hertig, D. Piasecka-Kwiatkowska, H. Staniek, K.W. Nowak, T. Twardowski: Study on iron availability from prepared soybean sprouts using iron-deficient rat model (DOC 57.0 kb) [file 11130_2014_413_MOESM1_ESM.doc]

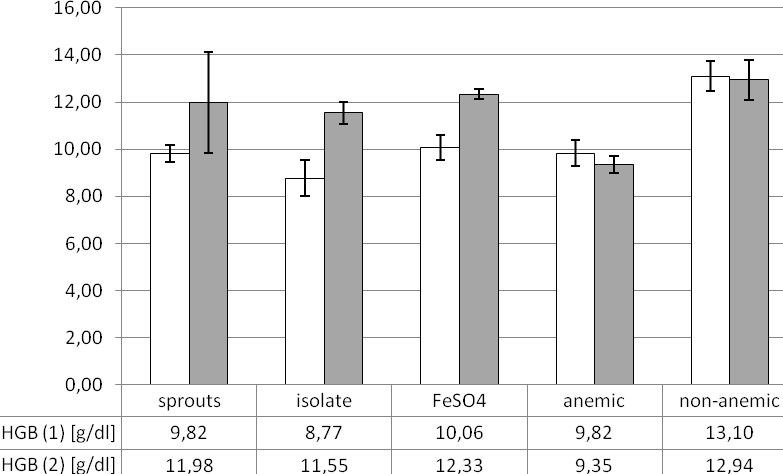


b

a

b

a

c

b

b

b

a

b

Electronic supplementary material Fig. 1. The changes in hemoglobin (HGB) concentration after supplementation of iron-deficient rat with ferritin-iron. The chart presents the hemoglobin concentration before (HGB1) and at the end (HGB2) of the experiment. Experimental group: ‘sprouts’ – iron deficient rats supplemented with soybean sprouts enriched in ferritin iron; ‘isolate’ - iron deficient rats supplemented with isolate of plant ferritin, ‘FeSO_4_’ – iron-deficient rats supplemented with pharmaceutical preparation – FeSO_4_, ‘anemic’ – control group of iron deficient rats during the whole experiment, ‘non-anemic’ - control group of healthy animals. *Published in: Food Chemistry, 2012, 135:2622-2627; M. Zielińska-Dawidziak, I. Hertig, D. Piasecka-Kwiatkowska, H. Staniek, K.W. Nowak, T. Twardowski: Study on iron availability from prepared soybean sprouts using iron-deficient rat model.*
