# Supplementary material for: Effect of Iron Status in Rats on the Absorption of Metal Ions from Plant Ferritin
Source: Plant Foods Hum Nutr. 2014 Apr 12;69(2):101–7. doi: 10.1007/s11130-014-0413-1 (PMC4032463; doi:10.1007/s11130-014-0413-1)
Supplement: Supplementary file 2 — Comparison of iron reserves in the liver and blood of experimental animals after supplementation with plant ferritin. Group of animals: iron deficient rats supplemented with soybean sprouts enriched in ferritin iron (1), iron deficient rats supplemented with isolate of plant ferritin (2), iron-deficient rats supplemented with pharmaceutical preparation (3), control group of iron deficient rats during the whole experiment (4), control group of healthy animals (5). Published in: Food Chemistry, 2012, 135:2622–2627; M. Zielińska-Dawidziak, I. Hertig, D. Piasecka-Kwiatkowska, H. Staniek, K.W. Nowak, T. Twardowski: Study on iron availability from prepared soybean sprouts using iron-deficient rat model (DOC 36.5 kb) [file 11130_2014_413_MOESM2_ESM.doc]

*Electronic supplementary material Table 1. Comparison of iron reserves in the liver and blood of experimental animals after supplementation with plant ferritin. Group of animals: iron deficient rats supplemented with soybean sprouts enriched in ferritin iron (1), iron deficient rats supplemented with isolate of plant ferritin (2), iron-deficient rats supplemented with pharmaceutical preparation (3), control group of iron deficient rats during the whole experiment (4), control group of healthy animals (5). Published in: Food Chemistry, 2012, 135:2622-2627; M. Zielińska-Dawidziak, I. Hertig, D. Piasecka-Kwiatkowska, H. Staniek, K.W. Nowak, T. Twardowski: Study on iron availability from prepared soybean sprouts* using iron-deficient rat model.

| No. | experimental group | Total iron content in the liver [mg] | ferritin content in the liver [mg] | Total iron content in the serum [µg/%] | Ferritin concentration in the serum [µg/ml] |
| --- | --- | --- | --- | --- | --- |
| 1 | Sprouted soybeen | 1.59  ±0.24 *a* | 15.84  ±3.15 *a.b* | 201  ±45 *b* | 2.62  ±0.33 *a. b* |
| 2 | Isolate | 1.80  ±0.21 *a* | 14.24  ±8.40 *a* | 187  ±18 *a.b* | 2.78  ±0.21 *b* |
| 3 | FeSO_4_ | 1.65  ±0.46 *a* | 8.44  ±3.37 *a* | 167  ±42 *a.b* | 2.24  ±0.26 *a* |
| 4 | Iron-deficient | 1.58  ±0.17 *a* | 8.82  ±1.40 *a* | 147  ±17 *a* | 2.23  ±0.28 *a* |
| 5 | Non-deficient | 2.27  ±0.69 *a* | 25.62  ±8.29 *b* | 204  ±39 *b* | 2.87  ±0.42 *b* |

Different letters in a column show statistically significant differences at *P* < 0.05
